# Navigating the Interplay of Sickle Cell Vasculopathy and Moyamoya Cerebrovascular Changes: A Case Report

**DOI:** 10.7759/cureus.67302

**Published:** 2024-08-20

**Authors:** Sabrina Carpintieri, Elias Uyar, Christian Anand, Yaroslav Buryk

**Affiliations:** 1 Medical School, Ross University School of Medicine, Miramar, USA; 2 Medical School, St. George's University School of Medicine, True Blue, GRD; 3 Pulmonary and Critical Care, Jackson Memorial Hospital, Miami, USA

**Keywords:** ischemic stroke, vaso-occlusive crisis (voc), chronic transfusion, antiplatelet therapy, multidisciplinary management, revascularization surgery, sta-mca bypass, cerebrovascular diseases, moya moya syndrome, sickle cell disease

## Abstract

Sickle cell disease (SCD) is a hereditary hemoglobinopathy that can lead to progressive vasculopathy, increasing the risk of cerebrovascular complications. Moyamoya syndrome (MMS), a rare disorder characterized by stenosis of the internal carotid arteries, can occur in SCD patients due to chronic endothelial damage and inflammation. The coexistence of these conditions can result in severe cerebrovascular complications, presenting unique diagnostic and therapeutic challenges. We present a 35-year-old African American male with a complex interplay of advanced SCD and MMS, manifesting as extensive cerebrovascular disease and recurrent ischemic strokes. A CT angiogram (CTA) of the head showed diffusely decreased caliber of the right M1 segment, appearing worse compared to prior studies. CTA of the head and neck demonstrated a new cut-off of the distal right M3 segment with an asymmetric paucity of arborizing vessels within the right middle cerebral artery (MCA) distribution, consistent with progressive sickle cell vasculopathy and also demonstrated abnormal dilated collateral vessels. Further imaging with MRI exhibited multiple prior ischemic strokes in various vascular territories despite previous revascularization surgery with a left superficial temporal artery to MCA bypass. The patient's progressive cerebrovascular disease was attributed to sickle cell vasculopathy exacerbated by MMS, resulting in compromised cerebral perfusion through distinct pathological mechanisms. Management involved a multidisciplinary treatment approach, including chronic transfusions, antiplatelet therapy, surgical revascularization with extracranial-intracranial bypass, seizure management, and neuropsychiatric support. Despite maximal therapy, the patient experienced recurrent cerebrovascular events and progressive neurological deficits, highlighting the challenges in controlling these intertwined disease processes. It signifies the importance of early recognition of this rare co-occurrence and implementation of prompt multidisciplinary treatment to improve outcomes.

## Introduction

Sickle cell disease (SCD) is a hereditary hemoglobinopathy characterized by the production of abnormal hemoglobin S. This leads to chronic hemolytic anemia and vascular occlusions, predisposing patients to progressive vasculopathy involving multiple organ systems [1]. Cerebrovascular disease is a major complication of SCD, with up to 24% of patients experiencing an overt stroke by adulthood [2]. Moyamoya syndrome (MMS) is a rare cerebrovascular disorder characterized by progressive stenosis of the terminal portions of the bilateral internal carotid arteries and their proximal branches. This leads to the development of an abnormal vascular network at the base of the brain, termed "moyamoya" vessels [3]. While the exact pathogenesis is unclear, it is thought to be caused by vascular endothelial damage and chronic inflammation [4].

In patients with SCD, chronic hemolytic anemia, vaso-occlusive crises, and release of vasculopathy factors can contribute to endothelial injury and inflammation, promoting the development of MMS [5]. Conversely, the progressive stenosis and compromised cerebral blood flow in MMS exacerbate ischemic injury in the setting of SCD vasculopathy [6].

We present a case of advanced SCD complicated by sickle cell anemia, recurrent vaso-occlusive crises, and the co-occurrence of MMS. This intricate interplay between the two disorders resulted in extensive cerebrovascular disease, recurrent ischemic strokes involving various cerebral territories [7], and progressive neurological deficits despite aggressive medical and surgical management. The case highlights the challenges in controlling disease progression when these two devastating conditions collide. Prompt recognition of this infrequent co-occurrence will help to rapidly initiate multidisciplinary treatment which will prevent further debilitating cerebrovascular complications in this critically ill patient population [8].

## Case presentation

The patient was a 35-year-old African American male with a longstanding history of SCD, frequent vaso-occlusive crises, major depressive disorder, focal to bilateral tonic-clonic seizures, and MMS status post left superficial temporal artery (STA) to middle cerebral artery (MCA) bypass a few years prior. His medication regimen included daily hydroxyurea to reduce the frequency of vaso-occlusive crises, folic acid supplementation to support red blood cell production, and non-steroidal anti-inflammatory drugs (NSAIDs) for pain management during less severe episodes. The patient's most recent hemoglobin electrophoresis showed an HbS% (sickle hemoglobin percentage) of 30%, a significant increase from 13% one month prior. This sharp rise in HbS% indicates a worsening of the patient's SCD. The increase suggests a suboptimal response to hydroxyurea therapy, possibly due to non-compliance with the prescribed treatment regimen. The high HbS% may have contributed to frequent crises and cerebrovascular complications, suggesting a need to reassess SCD management, including medication adherence and potential treatment adjustments. Imaging studies including MRI and CT angiography (CTA) demonstrated extensive cerebrovascular disease. He had a history of chronic transfusion regimen, left frontal craniotomy, and multiple ischemic strokes involving the left MCA territory and lacunar infarcts resulting in residual dysarthria and aphasia.

On admission, the patient presented with abdominal pain near his percutaneous endoscopic gastrostomy (PEG) tube site, similar to his prior sickle cell crisis pain. In the ER, he developed acute right-sided weakness and numbness, prompting a stroke alert. Imaging studies, including CT and MRI, revealed no acute findings but re-demonstrated extensive encephalomalacia in the left MCA territory, focal encephalomalacia in the right medial occipital lobe, and scattered periventricular and deep white matter hypodensities suggestive of microangiopathic ischemic changes. 

**Figure 1:**
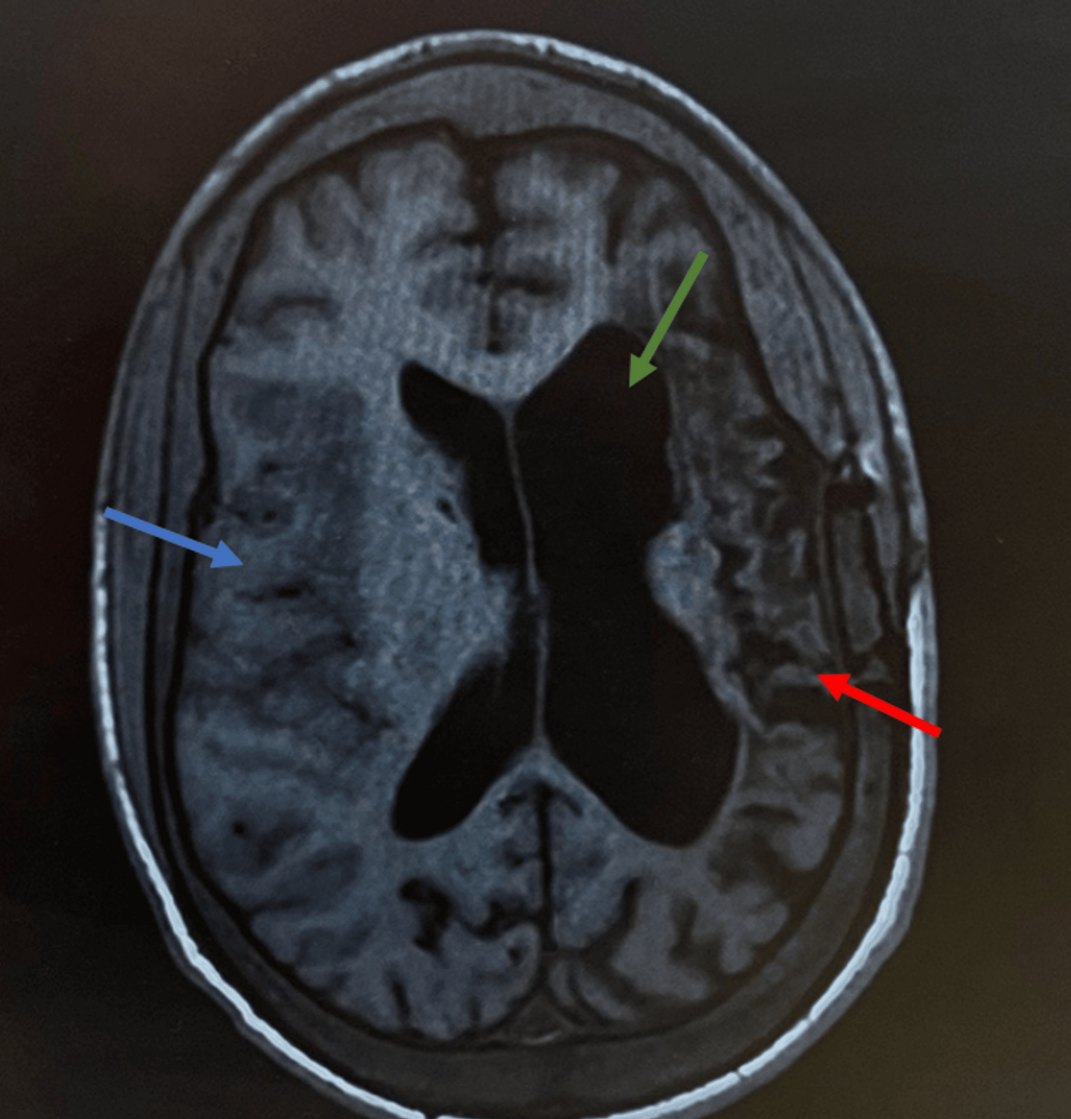
Non-contrast brain MRI showing multiple abnormalities The red arrow indicates extensive encephalomalacia involving the left hemisphere, with associated gliosis and hemosiderin staining. The blue arrow points to subtle hypodense changes in the posterior aspect of the right frontal lobe. The green arrow shows compensatory dilation of the left lateral ventricle.

The patient's condition was attributed to progressive sickle cell vasculopathy, and his anticoagulation was switched from apixaban to aspirin and cilostazol. A non-contrast brain MRI showed subtle hypodense changes in the posterior aspect of the right frontal lobe (Figure 1). There was extensive encephalomalacia (softening or loss of brain tissue) involving the left hemisphere, with associated gliosis and hemosiderin staining indicating prior hemorrhage. There was compensatory dilation of the left lateral ventricle, likely due to the tissue loss in the left hemisphere.

**Figure 2:**
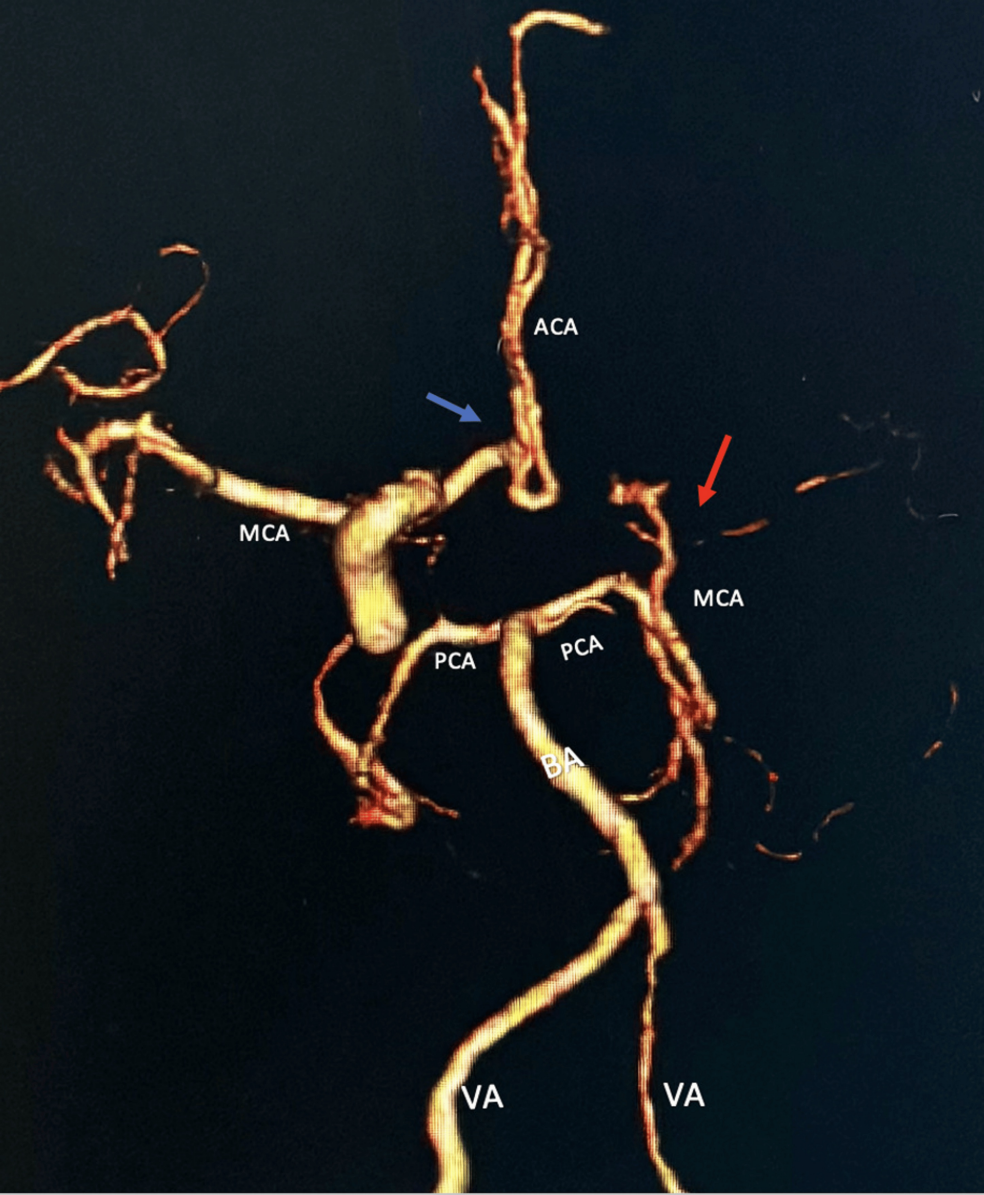
CTA of head showing a complete occlusion of the left M1 segment (red arrow), with minimal distant M2 and M3 branches visualized. The right MCA branches are unremarkable with mild narrowing of the proximal right A1 segment (blue arrow), and left ACA is poorly visualized. VA, vertebral artery; BA, basilar artery; PCA, posterior cerebral artery; MCA, middle cerebral artery; M1, first segment of MCA; M2, second segment of MCA; M3, third segment of MCA; ACA, anterior cerebral artery; A1, first segment of ACA; CTA: computed tomography angiography

A CTA of the head showed diffusely decreased caliber of the right M1 segment, appearing worse compared to prior studies (Figure 2). 

**Figure 3:**
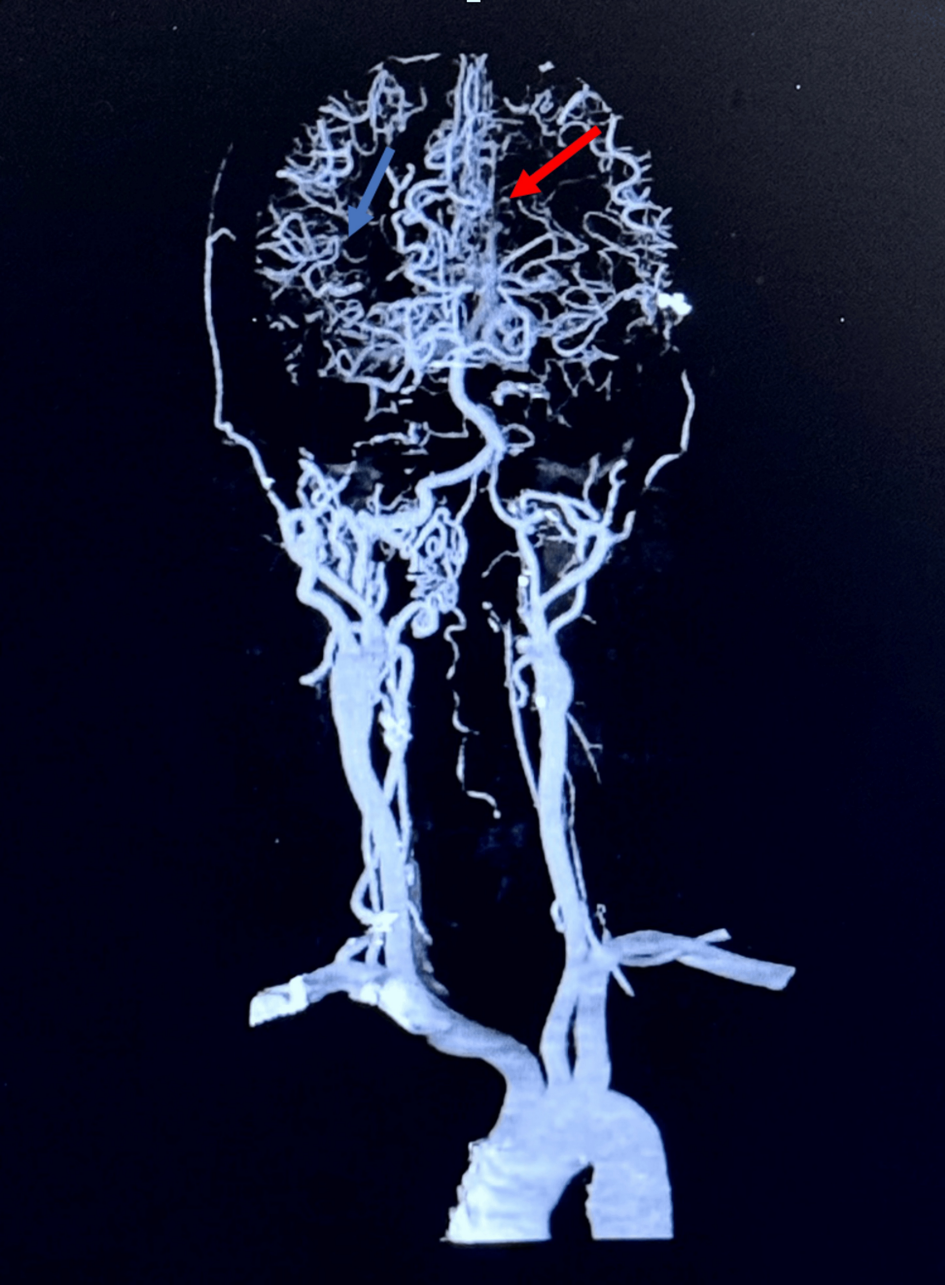
CTA of head and neck The red arrow highlights the "puff of smoke" or moyamoya pattern, with abnormal dilated collateral vessels forming in response to stenosis/occlusion of major cerebral arteries from the combined effects of sickle cell disease and moyamoya phenomenon. The blue arrow indicates the abrupt termination or cut-off of the distal right M3 segment of the middle cerebral artery due to vaso-occlusive changes from sickle cell disease. CTA: computed tomography angiography

The patient's speech worsened, and a CTA of the head and neck was performed confirming a new occlusion of the right M3 segment and asymmetric paucity of arborizing vessels within the right MCA distribution, consistent with progressive sickle cell vasculopathy, prompting a right-sided craniotomy and extracranial-intracranial bypass surgery. The CTA of the head and neck revealed an extensive network of small, tortuous collateral vessels, creating the characteristic "puff of smoke" appearance seen in MMS (Figure 3). 

In this patient's case, the compromised blood flow stemmed from two distinct pathologies: sickle cell vasculopathy and MMS. Sickle cell crises can precipitate vaso-occlusive narrowing or occlusion of the cerebral vessels, while MMS causes progressive stenosis of the terminal internal carotid arteries and their proximal branches. The combination of these two vascular conditions likely contributed to the extensive collateralization seen on imaging as the brain strived to maintain perfusion despite the multifocal vascular insults. The multidisciplinary treatment approach for this patient is outlined in Table 1.

**Table 1:** The multidisciplinary treatment approach NSAIDs: non-steroidal anti-inflammatory drugs; STA: superficial temporal artery; MCA: middle cerebral artery

Treatment	Description
Medical Management	Chronic transfusions, antiplatelet therapy (aspirin, cilostazol), anticoagulation (apixaban), hydroxyurea, folic acid, NSAIDs
Surgical Intervention	Left STA-MCA bypass (prior), right-sided craniotomy and extracranial-intracranial bypass
Supportive Therapy	Seizure management, neuropsychiatric support

## Discussion

Pathophysiological mechanisms 

This complex case exemplifies the intricate interplay between SCD and MMS, two distinct pathologies that can synergistically impair cerebrovascular integrity through separate mechanisms. SCD predisposes patients to chronic hemolytic anemia, vaso-occlusive crises, and progressive vasculopathy due to the production of abnormal hemoglobin S [2]. This vasculopathy can manifest in various ways and likely contributed to the development of MMS in this patient.

The pathophysiology behind the development of MMS in patients with SCD is not fully understood, but several mechanisms have been proposed. Chronic hemolytic anemia and vaso-occlusive episodes in SCD can lead to endothelial damage, chronic inflammation, and the release of vasculopathy factors, which may contribute to the progressive narrowing of the cerebral vasculature observed in MMS [5,9]. Additionally, the abnormal hemoglobin S can directly impair vascular endothelial function and promote vascular remodeling, further exacerbating the development of MMS [6]. Once established, the progressive stenosis in MMS can potentiate the deleterious effects of SCD on the cerebrovascular system. The progressive stenosis of the major cerebral arteries can lead to chronic cerebral hypoperfusion, increasing the risk of ischemic stroke and other cerebrovascular complications [7]. This vicious cycle can result in recurrent cerebrovascular accidents (CVAs), as illustrated in this case, where the patient experienced multiple ischemic strokes involving various cerebral territories, despite aggressive management.

Diagnostic modalities

The current case highlights the vital role of advanced imaging techniques in diagnosing and monitoring disease progression. CTA was invaluable for delineating the patient's extensive vasculopathy, including internal carotid artery occlusions, moyamoya changes, and progressive stenosis [7]. MRI elucidated the ischemic burden with multiple prior strokes across various vascular territories. Frequent serial imaging with CTA/magnetic resonance angiography (MRA) and brain MRI is necessary to detect new ischemic lesions and vascular changes to guide appropriate interventions [10].

Multidimensional treatment plans

The management of such a complex case requires a comprehensive multidisciplinary approach. Optimization of SCD management through chronic transfusions and prevention of vaso-occlusive crises is crucial. Surgical revascularization with procedures like superficial temporal artery (STA)-MCA bypass augments cerebral perfusion distal to stenoses [3]. Antiplatelet and antithrombotic agents reduce thrombotic risk and promote collaterals [11]. Seizure management, neuropsychiatric support, frequent multimodal imaging, and close multidisciplinary follow-up are also essential components [10].

Disease progression and outcomes 

This intricate pathophysiology culminated in extensive cerebrovascular disease in our patient, manifesting as multiple territory infarcts, internal carotid artery occlusions, and moyamoya vasculopathy despite previous revascularization attempts. His recurrent ischemic strokes and progressive deficits highlight the formidable challenges of controlling these interwoven disease processes, even with a multidimensional treatment strategy.

The patient's continued cerebrovascular events despite maximal therapy portray the limitations of current management for this co-occurrence. Ultimately, this case accentuates the need for increased clinical awareness of SCD predisposing to MMS. It illuminates gaps in understanding the exact mechanisms by which these diseases synergize and emphasizes the importance of developing novel therapeutic approaches. Prompt recognition allowing timely implementation of multidisciplinary care remains paramount to optimizing outcomes in this critically ill population at risk of devastating neurological sequelae.

Early recognition and prompt treatment are essential in reducing the risk of further cerebrovascular events and improving patient outcomes. Regular follow-up, adherence to treatment regimens, and a multidisciplinary approach involving hematologists, neurologists, neurosurgeons, and other specialists are crucial in managing these complex cases effectively.

## Conclusions

This case illustrates the devastating neurological consequences due to the complex interplay between SCD and MMS. Despite a comprehensive multidisciplinary treatment approach, the compounding effects of these two distinct cerebrovascular pathologies led to extensive neurovascular injury, recurrent ischemic strokes, and profound neurological deficits in this young patient. Enhancing awareness and education among healthcare professionals about this challenging combination of conditions is essential. Rapid diagnosis followed by prompt, coordinated multidisciplinary care may help reduce the risk of further cerebrovascular events and improve outcomes for these critically ill patients who face potentially severe neurological complications.

Continued research efforts are vital to better understand the precise pathophysiological mechanisms underlying the synergistic effects of these two conditions, which could lead to the development of novel, more targeted therapeutic strategies. Furthermore, the establishment of dedicated multidisciplinary teams or specialized centers for managing such complex cases could potentially improve coordination of care and ensure comprehensive, patient-centered management tailored to the unique needs of individuals afflicted by this challenging intersection.
